# Impact of feline AIM on the susceptibility of cats to renal disease

**DOI:** 10.1038/srep35251

**Published:** 2016-10-12

**Authors:** Ryoichi Sugisawa, Emiri Hiramoto, Shigeru Matsuoka, Satomi Iwai, Ryosuke Takai, Tomoko Yamazaki, Nobuko Mori, Yuki Okada, Naoki Takeda, Ken-ichi Yamamura, Toshiro Arai, Satoko Arai, Toru Miyazaki

**Affiliations:** 1 Laboratory of Molecular Biomedicine for Pathogenesis, Center for Disease Biology and Integrative Medicine, Faculty of Medicine, The University of Tokyo, Tokyo 113-0033, Japan; 2 Graduate School of Science, Osaka University, Osaka 560-0043, Japan; 3 Laboratory of Small Animal Surgery 2, School of Veterinary Medicine, Kitasato University, Aomori 034-8628, Japan; 4 Department of Basic Veterinary Medicine, School of Veterinary Medicine, Nippon Veterinary and Life Science University, Tokyo 180-8602, Japan; 5 Center of Advanced Studies in Veterinary Medicine, School of Veterinary Medicine, Nippon Veterinary and Life Science University, Tokyo 180-8602, Japan; 6 Center for Animal Resources and Development, Kumamoto University, Kumamoto 860-0811, Japan; 7 AMED-CREST, Japan Agency for Medical Research and Development, Tokyo 113-0033, Japan; 8 Max Planck-The University of Tokyo Center for Integrative Inflammology, Tokyo 113-0033, Japan

## Abstract

Renal failure is one of the most important social problems for its incurability and high costs for patients’ health care. Through clarification of the underlying mechanism for the high susceptibility of cats to renal disease, we here demonstrates that the effective dissociation of serum AIM protein from IgM is necessary for the recovery from acute kidney injury (AKI). In cats, the AIM-IgM binding affinity is 1000-fold higher than that in mice, which is caused by the unique positively-charged amino-acid cluster present in feline AIM. Hence, feline AIM does not dissociate from IgM during AKI, abolishing its translocation into urine. This results in inefficient clearance of lumen-obstructing necrotic cell debris at proximal tubules, thereby impairing AKI recovery. Accordingly, mice whose AIM is replaced by feline AIM exhibit higher mortality by AKI than in wild-type mice. Recombinant AIM administration into the mice improves their renal function and survival. As insufficient recovery from AKI predisposes patients to chronic, end-stage renal disease, feline AIM may be involved crucially in the high mortality of cats due to renal disease. Our findings could be the basis of the development of novel AKI therapies targeting AIM-IgM dissociation, and may support renal function in cats and prolong their lives.

The number of pets is increasing markedly worldwide alongside the recently decreasing birth rate and increasingly greater age of the human population, and cats are the most popular pet in the vast majority of areas^1^,^2^. It is well known that cats are profoundly more susceptible to and more often die from chronic kidney disease (CKD) than other animals^3^,^4^,^5^,^6^. However, the exact reason for their susceptibility to renal disease, which is one of the most pressing questions in veterinary medicine, remains unclear. Therefore, no effective therapies are available. In this study, we newly identified that feline apoptosis inhibitor of macrophage (AIM, also called CD5-like antigen [CD5L] and encoded by the gene *Cd5l*)^7^ protein remains inactive during acute kidney injury (AKI) in cats, and thus, is crucially involved in the susceptibility of cats to progressive renal disease. Furthermore, we propose that adequate resolution of acute injury by the administration of functional AIM may prevent progression to the lethal, chronic and end-stage renal disease.

AIM is a circulating protein present at a relatively high level in the blood^8^ that was initially identified as a supporter of macrophage survival^7^. AIM is produced by tissue macrophages under transcriptional regulation by nuclear receptor liver X receptor/retinoid X receptor heterodimers^9^,^10^,^11^. Under healthy states, AIM associates with IgM pentamers in the blood, which protects AIM from renal excretion and maintains high levels of circulating AIM^12^. AIM binds to multiple molecules including lipopolysaccharide^13^, cytoplasmic fatty acid synthase^14^, IgM^12^,^15^, and various regulators of complement activation. In addition, AIM is endocytosed by various cell types, including macrophages, adipocytes, and hepatocytes via scavenger receptors^14^,^16^,^17^,^18^. On the basis of these characteristics, we and others have demonstrated recently the involvement of AIM in the pathogenesis of a broad range of diseases including obesity^14^, insulin resistance^19^, atherosclerosis^9^,^20^, autoimmune diseases^12^, and non-alcoholic fatty liver diseases^8^,^16^,^21^. Most recently, we found that during AKI in humans and mice, AIM dissociates from IgM pentamers and appears in urine^22^. Urinary AIM accumulates at the lumen-obstructing necrotic cell debris at proximal tubules, and interacts with kidney injury molecule-1 (KIM-1; also known as TIM-1 or HAVCR1 and encoded by gene *Havcr1*) expressed on the injured tubular epithelial cells^23^,^24^,^25^. This response promotes the engulfment of intraluminal debris by the epithelial cells, thereby promoting AKI recovery^22^. Indeed, when subjected to ischemia/reperfusion (IR)-induced AKI, AIM-deficient (AIM^−^) mice exhibit abrogated debris clearance and persistent renal inflammation, resulting in higher mortality than wild-type mice due to progressive renal dysfunction. Treatment with recombinant AIM of mice with IR-induced AKI results in the removal of the debris, thereby ameliorating renal pathology^22^. Based on such important involvement of AIM in the resolution of AKI, we decided to evaluate feline AIM to assess whether it is related to the susceptibility of cats to renal disease.

## Results

### Isolation and functional characterization of feline AIM

**Figure 1:**
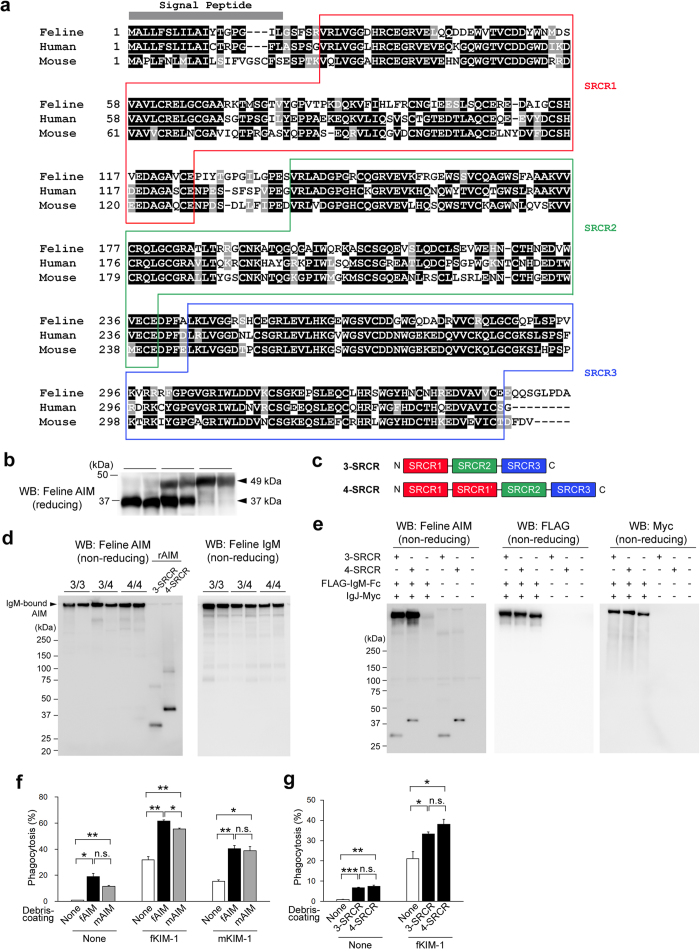
Identification of feline AIM. (**a**) Amino acid sequence alignment of feline AIM in comparison with human and mouse AIM. Residues with black background indicate identical amino acids and residues with grey background indicate similar amino acids. Three SRCR domains (SRCR1, 2, and 3) are indicated by coloured boxes. (**b**) Immunoblotting of cat plasma in a reducing condition using a newly generated anti-feline AIM polyclonal antibody. The arrows indicate 2 types of feline AIM. (**c**) Schema of the 3-SRCR and 4-SRCR feline AIM. (**d**) Immunoblotting in non-reducing conditions for AIM (left) and IgM (right) using plasma from cats possessing 3-SRCR (indicated by 3/3), 3/4-SRCR (indicated by 3/4), or 4-SRCR (indicated by 4/4) AIM. Recombinant proteins (20 ng each) of 3-SRCR and 4-SRCR AIM were loaded as controls. (**e**) HEK293T cells overexpressing 3- or 4-SRCR feline AIM and those overexpressing FLAG-tagged feline IgM-Fc and Myc-tagged feline IgJ were co-cultured for 16 h, and the supernatant was analysed for AIM by immunoblotting in non-reducing conditions. (**f**,**g**) *In vitro* phagocytosis assay. Engulfment of debris with rAIM coating by mProx24 cells overexpressing KIM-1 was assessed. The types of rAIM (mouse, 3-SRCR feline, or 4-SRCR feline) and KIM-1 (mouse or feline) are indicated. Engulfment of feline AIM (3-SRCR, indicated by fAIM) or mouse AIM (mAIM) coated debris by feline KIM-1(fKIM-1) or mouse KIM-1 (mKIM-1) expressing cells (**f**), and engulfment of 3-SRCR and 4-SRCR feline AIM coated debris by feline KIM-1 expressing cells (**g**). None: mProx24 cells without KIM-1 expression. The experiment was performed in triplicate, and the percentage (averages ± s.e.m.) of eFluor^®^780-positive mProx24 cells is shown as “Phagocytosis” on the graph.

Feline AIM cDNA was isolated from a cat spleen cDNA pool by 5′-rapid amplification of cDNA ends based on database sequence information of the predicted feline *AIM (cd5l*) mRNA. A comparison of the amino acid sequences of human, feline and mouse AIM is presented (Fig. 1a). The amino acid identity was at similar levels in feline and human AIM and in mouse and human AIM, which was 63.6% and 67.6%, respectively. Having generated several antibodies recognising feline AIM specifically, serum AIM levels were assessed by immunoblotting in >30 cats (strain information and AIM level of each animal are described in Supplementary Table 1). The overall average AIM level in cats was 21.2 μg/mL [8.48–36.39 μg/mL range in cats analysed], which is markedly higher than that in mice (2–3 μg/mL)^14^,^16^ and humans (approximately 5 μg/mL)^8^ (Supplementary Table 1). In blood, AIM associates with IgM pentamers; thus, serum levels of AIM and IgM are positively correlated in humans and mice^8^,^12^. However, as IgM levels in cats were found to be similar to those in humans and mice (1–2 mg/mL), the increased AIM levels in cats were not caused by higher IgM levels. Interestingly, immunoblotting of serum AIM revealed that cats could be classified into three groups by their AIM pattern: with only 37 kDa AIM, with 37 and 49 kDa AIM, and with only 49 kDa AIM (Fig. 1b). This difference was not due to variable glycosylation, as deglycosylation treatments of serum did not alter the pattern (Supplementary Fig. 1a). To this end, we analysed *AIM* mRNA from cats showing each serum AIM pattern, and found that 49 kDa AIM possessed 4 cysteine-rich (called scavenger receptor cysteine-rich [SRCR]) domains. Typically, AIM consists of 3 SRCR domains^7^, but feline 49 kDa AIM contained a duplicated first SRCR (SRCR1) domain (Fig. 1c). We also identified a minor variant of 3-SRCR and 4-SRCR feline AIM (one variant for each), in which the hinge region between SRCR1 and SRCR2 was variable (Supplementary Fig. 1b). Thus, blood AIM protein showing the size of 37 kDa only, 37 and 49 kDa, and 49 kDa only in immunoblotting represent 3-SRCR AIM homozygote, 3-/4-SRCR AIM heterozygote, and 4-SRCR AIM homozygote, respectively. Both 3-SRCR and 4-SRCR AIM are associated with IgM pentamers in blood, as shown by immunoblotting of the three types of cat sera in a non-reducing condition (Fig. 1d). This was corroborated by an *in vitro* association experiment using feline recombinant AIM (rAIM) and feline IgM Fc proteins (Fig. 1e). Note that we previously showed that AIM binds to the Fc region of IgM^12^,^26^.

The most notable AIM function in facilitating AKI repair is the enhancement of clearance of dead cell debris in the proximal tubules. During AKI, the cell death in the kidney occurs due to apoptosis and necroptosis, particularly in the proximal tubules at the corticomedullary junction, and such dead cells detach from the tubular basement membrane and physically obstruct the tubular lumen. These events reduce glomerular filtration and also induce the production of inflammatory mediators by injured epithelial and infiltrating hematopoietic cells, further exacerbating tubular injury and disturbing the tubular degeneration^27^,^28^,^29^. KIM-1, the expression of which is highly induced in tubular epithelial cells upon injury and is thus a well-known injury marker^23^,^24^,^25^, is a ligand for AIM, and induces the engulfment of AIM-deposited necrotic cell debris by tubular epithelial cells^22^,^30^. Therefore, we determined whether feline AIM may be deficient in accelerating phagocytosis of debris by feline KIM-1-expressing tubular epithelial cells, by performing an *in vitro* phagocytosis assay^22^. Debris was prepared from necrotic mProx24 cells, a mouse proximal tubular epithelial cell line, and then coated with feline recombinant AIM (rAIM) by co-incubation. The debris was incubated with living mProx24 cells overexpressing feline KIM-1. The effect of AIM coating on KIM-1-dependent phagocytosis was assessed quantitatively by flow cytometry. Debris engulfment by feline KIM-1-expressing cells increased significantly when debris was coated with feline rAIM and was at similar levels as observed when mouse AIM and mouse KIM-1 were used, suggesting that both feline AIM and feline KIM-1 were functional in promoting phagocytic action for necrotic cell debris (Fig. 1f). This effect was observed equivalently for 3-SRCR and 4-SRCR feline AIM (Fig. 1g). Interestingly, enhanced debris phagocytosis was also achieved at comparable levels for the combination of feline AIM and mouse KIM-1 or mouse AIM and feline KIM-1 (Fig. 1f). Thus, the collaborative function of feline AIM and KIM-1 in the enhancement of debris engulfment was essentially normal.

### Impaired recovery from AKI in AIM felinised mice

**Figure 2:**
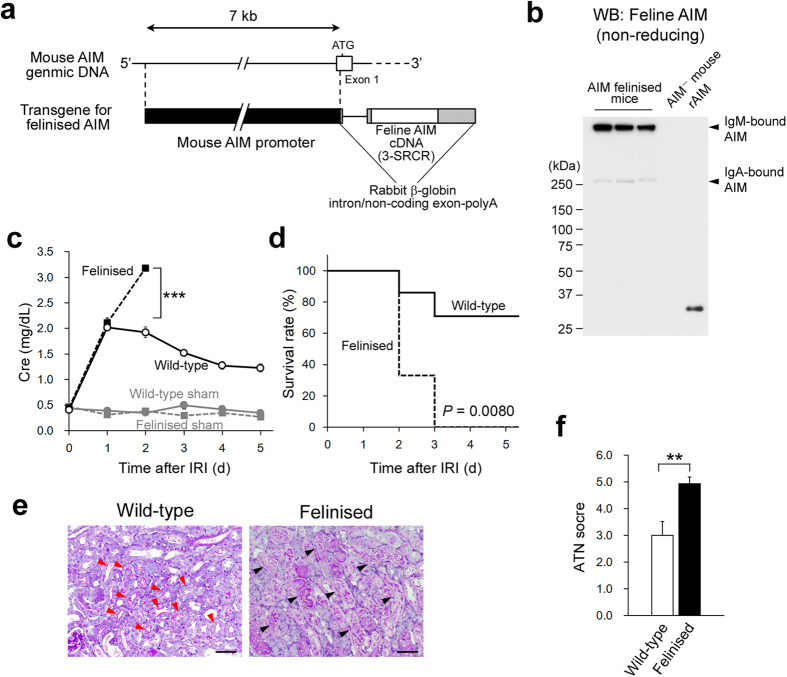
Impaired AKI repair in AIM felinised mice. (**a**) Schema of the transgene. (**b**) Immunostaining in a non-reducing condition for feline AIM involving sera from AIM felinised mice (n = 3). AIM^−^ mouse serum and 3-SRCR feline rAIM (10 ng) were loaded as a negative and a positive control, respectively. IgM-bound and IgA-bound transgenic feline AIM are indicated. (**c**) The Cre levels on the indicated days after IR. Open circles, wild-type mice (*n* = 7); closed squares, AIM felinised mice (*n* = 6). Sham control mice (*n* = 3 for each) were also analysed. Grey circles, sham wild-type mice; grey squares, sham AIM felinised mice. Male mice at 8–10 weeks of age were used. ***P < 0.001. (**d**) Survival of wild-type and AIM felinised mice after IR (Kaplan-Meier method). *P*: statistical significance between each type of mouse was calculated by using the log-rank test. Solid line, wild-type mice (*n* = 7); dotted line, AIM felinised mice (*n* = 6). (**e**) Representative PAS-stained histologic photomicrographs of the kidney from wild-type and AIM felinised mice (n = 3 for each group) on day 3 after IR. Photomicrographs of the corticomedullary junction area are presented. Red arrows: brush border; black arrows: intraluminal debris. Scale bar: 50 μm. (**f**) The ATN score in the kidney of IR-treated wild-type mice and AIM felinised mice on day 3 after IR. *n* = 3 each.

Then, to assess directly the impact of feline AIM in the poor prognosis of kidney disease in cats, we attempted to felinise AIM in mice and induce AKI. For this purpose, we generated transgenic mice expressing feline AIM under the regulation of the mouse AIM promoter on a mouse AIM^−^ background. Since the function of 3-SRCR and 4-SRCR feline AIM appeared to be comparable in debris clearance (Fig. 1g), we here employed 3-SRCR feline AIM for the transgene. AIM is produced by tissue macrophages under transcriptional regulation by nuclear receptor liver X receptor (LXR)/retinoid X receptor heterodimers^9^,^10^. As the LXR response element is located at position −5404 of the AIM promoter^10^, we used a genomic DNA fragment of approximately 7 kb upstream of the *AIM* gene to construct the transgene (Fig. 2a). We confirmed that the expression distribution of transgenic feline AIM was comparable to that of endogenous mouse AIM, which is dominant in the resident macrophage-rich liver and spleen^7^, by QPCR and immunohistochemistry (Supplementary Fig. 2a,b). Transgenic feline AIM protein associated with mouse IgM pentamers (Fig. 2b). Although AIM associates predominantly with IgM, it also binds to IgA^12^. In serum of AIM felinised mice, AIM was also detected in association with IgA (Fig. 2b). The serum levels of feline AIM in AIM felinised mice were comparable to those of mouse AIM in wild-type mice (approximately 2 μg/mL) when assessed by immunoblotting (Supplementary Fig. 2c).

Wild-type and AIM felinised mice were subjected to a standard ischemia/reperfusion (IR) protocol to induce AKI that is usually reversible in wild-type mice^22^. Serum CRE levels peaked at day 1 and recovered thereafter in wild-type mice (Fig. 2c) and 75% of mice survived at day 5 (Fig. 2d). In striking contrast, in AIM felinised mice, serum CRE increased to similar levels as in wild-type mice at day 1 after IR, but continued to increase thereafter (Fig. 2c), resulting in the death of 100% mice by day 3 (Fig. 2d).

One of the important pathological characteristics often observed in AKI is renal tubular obstruction by debris from dead tubular epithelial cells^27^,^28^,^29^. In histology, on day 3 after IR, there was significant less periodic acid-Schiff (PAS)-stained intraluminal debris at proximal tubules located at the corticomedullary junction in wild-type mice than in AIM felinised mice (Fig. 2e). Similarly, recovery of the brush border in the proximal tubules was apparent in wild-type mice but not in AIM felinised mice (Fig. 2e). The overall acute tubular necrosis (ATN) score^31^ at day 3 was significantly worse in AIM felinised mice than in wild-type mice (Fig. 2f). Thus, AIM felinised mice showed impaired recovery from AKI.

### Feline AIM does not dissociate from IgM during AKI

**Figure 3:**
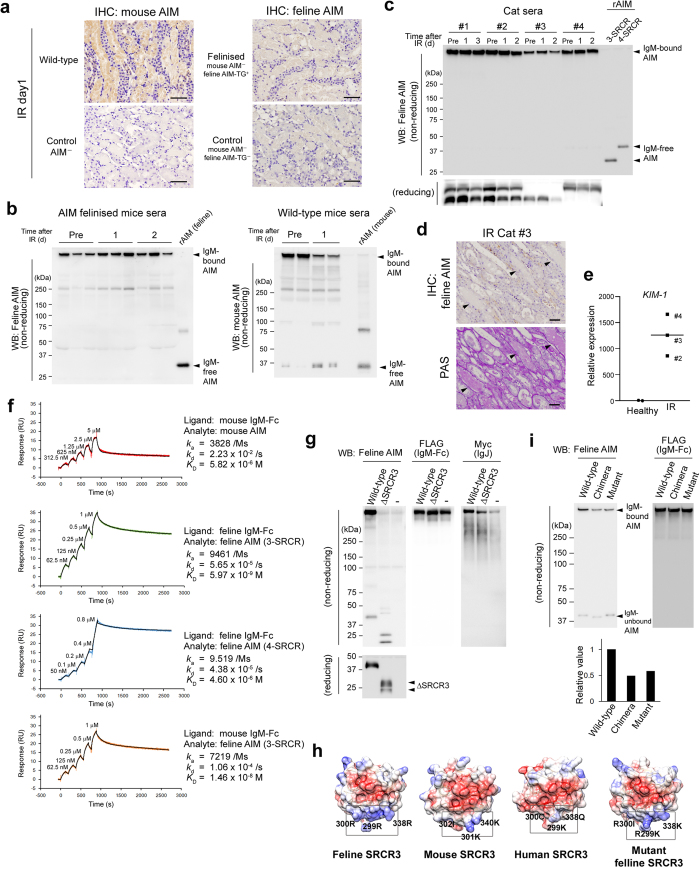
No dissociation of feline AIM from IgM pentamer. (**a**) Immunohistostaining of the kidney at day 1 after IR. Kidney specimens of AIM^−^ mouse were also immunostained for mouse or feline AIM. Signals were visualised by HRP/DAB. Five mice each were analysed. (**b**) Immunoblotting in non-reducing conditions for feline AIM (AIM felinised mice; n = 3), and mouse AIM (wild-type mice; n = 2). Pre: before IR. 3-SRCR feline rAIM (15 ng) and mouse rAIM (20 ng) were loaded as controls. (**c**) Immunoblotting of 4 IR cats in a non-reducing (upper panel) or reducing (lower panel) condition for AIM. Genotype: #1 and 2, 3/4-SRCR; #3, 3/3-SRCR, #4, 4/4-SRCR. rAIM (15 ng each) were loaded as controls. A longer-exposed blot is in Supplementary (**b**). (**d**) Immunostaining for feline AIM at the corticomedullary junction in the kidney of an IR cat (#3) at day 2. Scale bars, 50 μm. Black arrows: intraluminal debris. Results from other IR cats are in Supplementary (**d**). (**e**) Relative *KIM-1* mRNA levels in the kidney of 3 cats after IR. Kidneys from healthy cats (26 months of age, male, n = 2) were analysed as controls. (**f**) SPR analysis for AIM (as analyte) and IgM pentamers (as ligand). Three sets of experiments were performed and similar results were obtained. (**g**) Immunoblotting in a non-reducing (upper panel) or reducing (lower panel) condition for feline AIM. Two different sizes of ΔSRCR-3 are indicated by arrows. Wild-type: wild-type feline AIM (3-SRCR). (**h**) Three-dimensional mapping of the charge distribution of amino acids for the SRCR3 domain. Positively charged: blue; negatively charged: red; neutrally charged: white. The feline AIM-specific cluster of positively charged amino acids is gated. Feline-specific arginine residues, and the corresponding amino acids in mouse, human and mutant feline AIM, are demonstrated. (**i**) Binding of wild-type, chimeric, and mutant feline AIM with feline IgM Fc pentamer assessed by immunoblotting (non-reducing conditions). Signal intensities of the IgM-Fc bound AIM were quantified using NIH ImageJ software, and the relative values to that in wild-type feline AIM are presented in a graph.

During AKI, serum AIM dissociates from IgM pentamer and the IgM-free AIM translocates to urine. The urinary AIM accumulates at intraluminal debris, thereby promoting its clearance^22^. To our surprise, immunohistochemical analysis of the kidney from IR-AIM felinised mice revealed that the intraluminal debris was not stained for AIM, in sharp contrast to the AIM-positive debris in wild-type mice, suggesting that AIM did not appear in urine after IR in AIM felinised mice (Fig. 3a). In support, immunoblotting of sera showed that IgM-free AIM was not detected after IR in AIM felinised mice, as observed in cats, whereas it was increased in wild-type mice (Fig. 3b). Thus, it appeared that feline AIM did not dissociate from IgM pentamer upon AKI induction.

We therefore assessed IgM-free AIM in sera of 4 cats with AKI artificially induced by IR^32^ at different injury levels. The information of the AKI cats is presented in Supplementary Table 2. All IR cats showed poor recovery from AKI, as determined by the serum levels of creatinine (Cre) and blood urea nitrogen (BUN) (Supplementary Fig. 3a). Immunoblotting of sera from mice with AKI in non-reducing conditions shows substantially increased amounts of IgM-free AIM (<40 kDa) with decreased IgM-bound AIM (>600 kDa) compared to those of non-AKI mice (Fig. 3b right, also shown in ref. 22). In contrast to this observation, sera from cats with AKI had no IgM-free AIM. Note that a small amount of IgM-free 4-SRCR AIM was detected even before IR (in #1, 2 and #4 cats), but its level did not increase upon IR (Fig. 3c, a longer-exposed blot is presented in Supplementary Fig. 3b). In addition, we examined urine from IR AKI-cats for the presence of AIM using an enzyme-linked immunosorbent assay (ELISA). In humans and mice, AIM is not detectable in urine samples from healthy individuals, and its levels increase under AKI^22^. Consistent with the serum results above, AIM was not detectable in urine of IR AKI-cats, indicating no urinary AIM excretion under AKI (Supplementary Fig. 3c). Furthermore, histological analysis demonstrated no AIM accumulation in the intraluminal debris of cats after IR (Fig. 3d, Supplementary Fig. 3d), in contrast to humans and mice (Fig. 3a left) with AKI where AIM accumulation is observed in debris^22^. Moreover, we analysed sera from several hospitalized cats with spontaneous AKI caused by various events such as renal stones. Again, no IgM-free AIM was observed in serum from these cats (Supplementary Fig. 3e). Thus, in cats, it is likely that feline AIM did not dissociate from IgM pentamers adequately during AKI and was not excreted in urine, causing less sufficient recovery from AKI due to inefficient intraluminal debris clearance. It is noteworthy that *KIM-1* mRNA expression was highly increased in the kidney in IR-cats, as assessed by quantitative PCR (QPCR) (Fig. 3e). Importantly, *KIM-1* mRNA levels continued to increase in all IR cats (Fig. 3e), in accordance with the observations that the serum levels of Cre and BUN did not improve after IR (Supplementary Fig. 3a). This is similar to the situation in AIM felinised mice (and also AIM^−^ mice^22^) after IR, in contrast to wild-type mice, where these elements improve promptly.

### Extremely high binding affinity of feline AIM with IgM

To obtain a mechanistic view for the *undissociativeness* of feline AIM from IgM, we employed surface plasmon resonance (SPR) analysis to quantitatively compare the binding affinity constant of AIM/IgM-Fc pentamers in feline and mouse AIM. The resulting association rate constant (*k*_a_) was 9461/molar second (Ms), the dissociation rate constant (*k*_d_) was 5.65 × 10^−5^/s, and the dissociation-association rate (*K*_D_) was 5.97 × 10^−9^ M in feline AIM (3-SRCR)/IgM-Fc, whereas in mouse AIM/IgM-Fc they were 3828/Ms, 2.23 × 10^−2^/s, and 5.82 × 10^−6^ M (Fig. 3f). Thus, in cats, AIM associates with IgM approximately 1000-fold more strongly than in mice. Interestingly, the 4-SRCR feline AIM and IgM-Fc, the *k*_a_ (that shows the binding efficient) was markedly lower (9.519/Ms) compared to that for 3-SRCR feline AIM and IgM-Fc (9461/Ms), whereas the *k*_d_ (that shows the dissociation efficiency) was high (4.38 × 10^−5^/s), comparable to that for 3-SRCR feline AIM/IgM-Fc (5.65 × 10^−5^/s) (Fig. 3f). This result suggests that 4-SRCR feline AIM is less efficient in binding with IgM-Fc than 3-SRCR feline AIM, but its dissociation from IgM may be as inefficient as that of 3-SRCR feline AIM. Therefore, it is likely that 4-SRCR feline AIM may not be released efficiently upon AKI. This result is consistent with the finding that a level of IgM-free 4-SRCR AIM was detected in non-AKI cat serum, but it did not increase upon AKI induction (Fig. 3c, Supplementary Fig. 3b). Since the binding affinity constant between feline AIM and mouse IgM-Fc pentamers was also high (Fig. 3f), it is likely that the strong association was brought about by feline AIM, and not by feline IgM. This is consistent with the observation that feline AIM was not released from mouse IgM pentamer in AIM felinised mice with AKI (Fig. 3b). It is also possible that the strong binding of feline AIM with IgM might contribute partly to the increased serum AIM levels in cats.

In addition, we found that the third SRCR domain (SRCR3) appeared to be responsible for IgM binding, since its deletion abrogated the association with IgM (Fig. 3g; similar results for mouse and human AIM will be published elsewhere). In particular, three-dimensional mapping of the charge distribution of amino acids revealed that SRCR3 domain of feline AIM possessed a cluster of positively charged amino acids (indicated by blue) on the surface of the molecule, which was not observed in mouse and human AIM (Fig. 3h, gated area). Thus, it is possible that the feline AIM-specific cluster of positively charged amino acids might be involved in the increase its binding affinity with IgM. This is reminiscent of the fact that complement C1q also binds to the Fc region of IgM, and the positively charged amino acids of C1q are involved in its association with IgM^33^. To test this hypothesis, we created a chimeric feline AIM in which SRCR3 was substituted to that of mouse AIM, and, furthermore, a mutant feline AIM in which feline-specific arginine residues consisting the positively charged amino acid cluster (at 299, 300, and 338 in Fig. 1a, Supplementary Fig. 3f) were substituted to the corresponding amino acids of mouse sequence, and verified their binding affinity with feline IgM-Fc. Intriguingly, although we first performed SPR analysis, both chimeric and mutant feline AIM showed a high level of non-specific binding to the surface of the SPR sensor chip, which was not observed for wild-type feline or mouse AIM. This artificial response interfered with the SPR quantification of the AIM/Fc binding affinity (Supplementary Fig. 3g). Therefore, instead, we evaluated their binding biochemically, by immunoblotting the IgM Fc-bound AIM in the supernatant after a co-culture of feline Fc-expressing HEK293T cells, and cells expressing either wild-type, chimeric, or mutant feline AIM. Both chimeric and mutant feline AIM exhibited a decreased binding to the Fc pentamers, suggesting that the positively charged amino acid-cluster in feline SRCR3 domain is responsible to the increase in the binding affinity of feline AIM and IgM-Fc (Fig. 3i).

### Administration of rAIM facilitates recovery from AKI in AIM felinised mice

**Figure 4:**
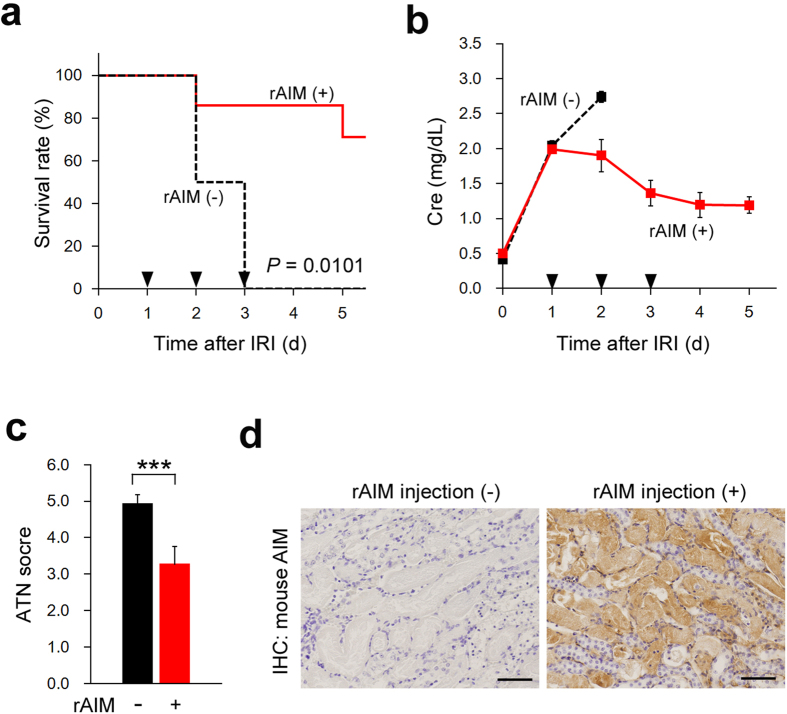
Amelioration of AKI in AIM felinised mouse by rAIM. (**a**) Survival of AIM felinised (*n* = 4, black dotted line) and mouse rAIM-injected AIM felinised mice (*n* = 7, red solid line) after IR (Kaplan-Meier method). ▼; rAIM (mouse; 1 mg) injected. *P*: statistical significance between each type of mouse was calculated by using the log-rank test. (**b**) The Cre levels on the indicated days after IR. Black squares, AIM felinised mice (*n* = 4); red squares, rAIM (mouse; 1 mg)-injected AIM felinised mice (*n* = 7). Male mice at 8–10 weeks of age were used. (**c**) The ATN score in the kidney of IR-treated AIM felinised mice (*n* = 3) and rAIM-injected AIM felinised mice (*n* = 3) on day 3 after IR. (**d**) Accumulation of AIM on intraluminal debris in rAIM-injected AIM felinised mice at day 1 after IR. Signals were visualised by HRP/DAB. Scale bars, 50 μm.

Lastly, we tested whether the administration of rAIM facilitates AKI recovery in AIM felinised mice. We injected mouse rAIM intravenously into AIM felinised mice on days 1, 2 and 3 after IR. The survival of rAIM-injected mice was improved markedly to 80% (Fig. 4a). The serum Cre level in rAIM-injected mice decreased by day 5, whereas in non-treated AIM felinised mice, it continued to increase until the mice died (Fig. 4b). By histology, the ATN score of rAIM-injected AIM felinised mice was improved at day 5 (Fig. 4c). We also confirmed by immunohistochemistry that injected rAIM accumulated at intraluminal debris (Fig. 4d). Thus, rAIM administration ameliorated AKI in AIM felinised mice, implying its therapeutic potential in cats.

## Discussion

Of the various important findings obtained in the current study, the evidence suggesting the linkage between the unique characteristics of feline AIM and the impaired recovery from AKI in cats may be especially noteworthy (Summarized in Supplementary Fig. 4). This finding strongly supports that the effective dissociation of AIM during AKI is necessary for recovery from AKI. Feline AIM was not released from IgM pentamers during AKI, and therefore, did not appear in urine. One might argue that the mechanism responsible for AIM dissociation from IgM, which remains unknown, might be defective in cats. This is not likely, however, because feline AIM did not dissociate from IgM in mice with AKI as shown in AIM felinised mice, where the releasing mechanism must be active. Certainly, however, we cannot exclude a possibility that feline AIM might exhibit not only increased binding affinity to IgM, but also unresponsiveness to the releasing mechanism. If so, it will be interesting to determine whether the both characteristics of feline AIM are brought about by feline SRCR3 domain, particularly by the unique cluster of positively charged amino acids. Future clarification of the releasing mechanism will answer this question.

Although the transition from AKI to CKD is still controversial^34^,^35^,^36^,^37^, AKI certainly predisposes patients to CKD development^38^,^39^,^40^,^41^,^42^,^43^. Thus, inadequate recovery of AKI due to feline AIM must be an important risk factor for the high susceptibility of cats to CKD. In support of this hypothesis, we observed in a previous study that after mild IR, AIM^−^ mice (whose kidney phenotype during AKI is essentially the same with that in AIM felinised mice) showed chronically persisting inflammation and progressive fibrosis, as well as higher *KIM-1* mRNA levels in the kidney compared to wild-type mice^22^. Of note, many primary diseases that cause cat CKD have the properties of AKI-causing incidents such as renal stones, infectious nephritis and peritonitis, and exposure to renal toxins including some antibiotics and anti-inflammatory reagents^44^,^45^,^46^. In this aspect, AIM administration during such acute events would be a potential therapy to prevent CKD in cats. Certainly, however, a proportion of cat CKD may occur without a preceding history of AKI. For instance, obesity and obesity-associated diseases including diabetes are also risk factors for CKD, and in fact, the prevalence of obesity is increasing rapidly in cats^47^,^48^,^49^. AIM also possesses an anti-obesity effect: IgM-free AIM is incorporated into adipocytes and induces lipolysis by reducing the enzymatic activity of fatty acid synthase within the cells^14^,^17^. Indeed, in AIM^−^ mice fed a high-fat diet, hypertrophy of adipocytes occurs more abundantly and the mass of visceral adipose tissues is greater, leading to more advanced obesity than in wild-type mice^14^. Thus, it is possible that feline AIM, which is characterised by its less efficient release from IgM, may also predispose cats to obesity, thereby contributing to CKD development. In this regard, AIM administration might also be effective for CKD prevention indirectly by suppressing the progression of obesity. Certainly, it is worthwhile to assess whether AIM administration would be directly effective for the amelioration of AKI-independent CKD. In conclusion, in addition to our previous study in AKI mice, our current findings may further corroborate the impact of AIM and its efficient release from IgM on protection from kidney disease, and thus, support its future therapeutic availability in humans. Moreover, our study provides a basis for the development of a next-generation therapy against renal failure in cats that might support their health and longevity.

## Methods

### General experimental approaches

No samples, mice, or data points were excluded from the reported analyses. The samples were not randomised to experimental groups. Analyses were not performed in a blinded fashion, except the evaluation of intraluminal debris and ATN score based on histologic results.

### Mice

AIM^−^ mice^7^ had been backcrossed to C57BL/6 (B6) for 15 generations before they were used for experiments. All animal experiments were carried out in strict accordance with the recommendations in the Guide for the Care and Use of Laboratory Animals of the National Institutes of Health. The protocol was approved by the Committee on the Ethics of Animal Experiments of the University of Tokyo (Permit Number: P10-143). All surgeries were performed under sodium pentobarbital anaesthesia, and all efforts were made to minimise suffering.

### Cat samples and experiments

Serum and plasma samples were collected from healthy cats after obtaining informed consent in writing from their owners. The cat IR experiments were performed under anaesthesia, and all efforts were made to minimise suffering. The study protocol was approved by the Ethics Committee of the Nippon Veterinary and Life Science University (Permission Numbers: #27-1 & #s27-1), and of Kitasato University (Permission Number: #15-056).

### Antibodies and reagents

The antibodies and reagents used for histology and biochemical experiments are as follows. Primary antibodies specific for mouse AIM (rab2 rabbit polyclonal) and feline AIM (PAC11 rabbit polyclonal, clone #33 and #56 mouse monoclonal) were established in our laboratory. Antibodies for cats were generated by immunizing mice (monoclonal) or rabbits (polyclonal) with feline AIM recombinant proteins tagged with an HA peptide. PAC-11 was used for immunoblotting in reducing conditions, whereas #33 and #56 were used for immunoblotting in non-reducing conditions, immunohistochemistry, and ELISA. We also used antibodies against mouse IgM (polyclonal; Santa Cruz Biotechnology), cat IgM (goat polyclonal; Bethyl Laboratories), HA (clone: 3F10; Roche), FLAG (clone: M2; SIGMA), and c-myc (goat polyclonal; QED Bioscience Inc). In addition, goat anti-rabbit IgG (H+L) secondary antibody, HRP conjugate (Thermo Fisher Scientific), were used. Cat serum IgM was quantified using a Cat IgM ELISA Quantification Set (Bethyl Laboratories, Inc., Montgomery, USA).

### Cloning of feline AIM cDNA

The 5′-cDNA was synthesised from a cat spleen RNA pool using a SMARTer^TM^ RACE cDNA Amplification Kit (Clontech Lab., Inc.). The 5′-cDNA of feline AIM was amplified using the Universal Primer A Mix supplied with the kit and a reverse primer (fSRCR1_rev) designed for SRCR1 of the predicted feline *cd5l* mRNA sequence (XM_011291200.1; GI:755808687). After obtaining the correct sequence of the 5′-end of feline *AIM* mRNA, we then amplified full-length feline AIM cDNA from a cat tissue cDNA pool (purchased from Zyagen Laboratories, Inc., CA) by PCR. By this method, 3-SRCR feline AIM cDNA was obtained. It is suspected that the cat cDNA pool used was from a 3-SRCR AIM homozygote cat (or cats). The 4-SRCR feline AIM cDNA was isolated by RT-PCR using splenic RNA isolated from a cat that possessed 4-SRCR AIM in serum. PCR primers were designed for SRCR1 (including ATG) and SRCR3 (including TAG) according to the sequence of 3-SRCR feline AIM (fAIM_5_f_EcoRI and fAIM_3_r_XhoI). Sequences of the oligonucleotides used are presented in Supplementary Table 4.

### SPR analysis

The interaction of AIM proteins with immobilised IgM-Fc pentamers were examined at 25 °C using a Biacore T100 SPR instrument (GE Healthcare, Little Chalfont, UK). IgM-Fc pentamers were covalently immobilised on a CM5 sensor chip (GE Healthcare) at pH 4.5 to 1000 resonance units using the amine coupling procedure at a flow rate of 10 μL/min. A reference flow cell, on which BSA (albumin, bovine, fraction V; Sigma) was immobilised, was used to record the background response, which was subtracted from each sample. Measurements were performed according to single-cycle kinetic analysis. Recombinant AIMs were diluted in a running buffer (Dulbecco phosphate-buffered saline [D-PBS (−)] pH 7.4; Nacalai Tesque) at a concentration ranging from 50 nM to 5 μM, and injected into flow cells at a flow rate of 30 μL/min for 120 sec repeating five times at 60-sec intervals. After injection, AIMs were allowed to dissociate in the running buffer for 30 min. The results were analysed using BIAcore T100 evaluation software. The differences in binding responses on the IgMFc-immobilised flow cell and the control BSA flow cell were fit to the heterogeneous ligand model to determine kinetic and affinity constants.

### Homology modeling

Homology models of AIM SRCR3 domains were generated using the Swiss-Model server ( http://swissmodel.expasy.org/SWISS-MODEL)^50^,^51^. The SWISS-MODEL template library (SMTL version 2016-04-06, PDB release 2016-04-01) was searched with Blast^52^ and HHBlits^53^ for evolutionary related structures matching the target sequences. The template with the highest quality (5a2e.1.A; PDB ID: 5A2E) was selected for model building. Models were built based on target-template alignment using Promod-II^54^. Coordinates which were conserved between the target and template were copied from the template to the model. Insertions and deletions were remodelled using a fragment library. Side chains were then rebuilt. Finally, the geometry of the resulting model was regularised by using a force field. When loop modelling with ProMod-II did not give a satisfactory result, an alternative model was built with MODELLER^55^. The global and per-residue model quality was assessed using the QMEAN scoring function^56^. The obtained QMEAN4 scores were −1.95, −2.36, −2.34, and −2.36 for feline, mouse, human, and mutant feline models, respectively. Molecular graphics images were produced using the UCSF Chimera package^57^ from the Computer Graphics Laboratory, University of California, San Francisco (supported by NIH P41 RR-01081).

### Induction of AKI in mice

IR induction was performed as described previously^22^,^58^. Briefly, mice (male, 8–12 weeks old) were anaesthetised by intraperitoneal Avertin injection (250 μg/kg bodyweight), and both kidneys were exposed through a small flank incision. Both renal arteries and veins were occluded with clamps at 37 °C. After the ischaemic period, the clamps were released to induce blood reperfusion. The number of mice tested was chosen to be sufficient to ensure statistical analysis of intra- and inter-group comparisons.

### Induction of AKI in cats

Cat IR was performed based on an established protocol for mouse IR after approval from the Ethics Committee of the Kitasato University, Japan. A recent report by Schmiedt *et al*.^32^ was also referred to. Briefly, the cats were anaesthetised by subcutaneous injection of atropine (0.05 mg/kg bodyweight) and intravenous injection of propofol (10 mg/kg bodyweight; Mylan Pharmaceutical Co., Ltd., Tokyo) followed by inhalation of isoflurane during the operation. The cats were boarded in the supine position, and both kidneys were exposed through an incision at the xiphoid to umbilicus midline. Both renal arteries and veins were separated from the surrounding tissue and occluded with Satin ski forceps for 1 h. After the ischemic period, the clamps were released to induce blood reperfusion. Ampicillin (20 mg/kg bodyweight) was administered intravenously before and after the operation. Occasionally, intravenous administration of buprenorphine (20 μg/kg bodyweight) was used for pain management. After the experiments, the cats were euthanised by intravenous injection of pentobarbital sodium (75 mg/kg bodyweight) and a saturated amount of potassium chloride.

### Evaluation of renal function in mice and cats

Serum mouse Cre concentrations were measured by using a Lab-Assay^TM^ Creatinine Kit (Wako Pure Chemical Industries, Ltd., Osaka, Japan). BUN and Cre levels were analysed using the SIMENS Dimension RXL Max System (SIEMENS Co., Ltd.) or JCA-BM2250 (JEOL Ltd., Tokyo, Japan). The mice were selected for blood drawing in a randomised manner.

### Histology

*PAS staining*: cat kidneys were fixed in 10% neutral-buffered formalin for 48 h, and mouse kidneys were fixed in 4% paraformaldehyde (PFA) in PBS for 24 h, followed by embedding in paraffin. PAS staining was performed on 4-μm sections of paraffin-embedded kidney blocks. *AIM detection on intraluminal debris*: 4 μm-sections of PFA-fixed paraffin-embedded kidney were immunostained with a rabbit anti-AIM polyclonal antibody (rab2) for mice or an anti-feline AIM monoclonal antibody (#33) for cats, followed by incubation with Histofine Simple Stain Mouse MAX-PO (R) (NICHIREI, Japan) for 30 min (for mouse specimens) or with Histofine Simple Stain MAX-PO (MULTI) (NICHIREI, Japan) for 30 min (for cat specimens). A Histofine Mouse Stain Kit (NICHIREI, Japan) was also used to detect cat AIM in felinised mouse tissues. After staining with DAB, the sections were counter-stained with hematoxylin. The specimens were subjected to analysis using a inverted microscope (FSX-100; Olympus).

### Evaluation of renal damage

The kidney specimens were stained with PAS, and PAS-stained debris (at corticomedullary junction) or brush border (corticomedullary junction and cortex region) were quantified by using NIH ImageJ software, and their percentage areas per whole section in a slide are presented. Five different sections of the kidney from at least 3 different mice of each group were examined. The ATN score was graded by proximal tubule dilation, brush border damage, proteinaceous casts, interstitial widening, and necrosis (0, none; 1, <11%; 2, 11–25%; 3, 26–45%; 4, 46–75%; 5, >75%)^22^. Evaluation of the specimens was performed in a blinded manner to mouse strain.

### Quantitative PCR assay

Quantitative evaluation of mRNA was performed by the ΔΔC_T_ method using a QuantStudio 3 Real-Time PCR system (Invitrogen; CA, USA). Sequences of the oligonucleotides used are presented in Supplementary Table 4.

### Production of recombinant proteins

For mouse AIM (mAIM), HEK293T cells were transfected with the pCAGGS-mAIM plasmid and cultured in DMEM + GlutaMax medium (Gibco, CA) supplemented with 5% foetal bovine serum (FBS) for 3 days. rAIM was purified from culture supernatant using a rat anti-mouse AIM monoclonal antibody (clone #36, made in house) -conjugated HiTrap NHS-activated HP column (GE Healthcare Life Sciences, PA). Bound protein was eluted with 0.1 M glycine-HCl pH 2.3 and neutralised with 1 M Tris-HCl pH 8.5. Protein was concentrated using Amicon Ultra filter concentrators (Millipore, MA), and stored at −80 °C in PBS. Endotoxin levels were measured using Limulus Color KY Test Wako (Wako Pure Chemical Industries, Ltd., Osaka). Protein concentration was determined by a bicinchoninic acid (BCA) assay according to the manufacturer’s protocol (Pierce, Rockford, IL). Recombinant feline AIM and AIM-HA were produced essentially by the same protocol except as follows. The pCAGGS-feline AIM and pCAGGS-feline AIM-HA plasmids were used; a mouse anti-feline AIM monoclonal antibody (clone #33) was used for purification of feline AIM, and bound protein was eluted with 0.1 M glycine-HCl pH 2.0; anti-HA matrix (Roche) was used for feline AIM-HA purification.

Feline and mouse Fc pentamers were produced as follows. HEK293T cells were co-transfected with pCAGGS-FLAG-IgM-Fc and pCAGGS-IgJ-Myc plasmids, then cultured in DMEM+GlutaMax medium (Gibco, CA) with 5% FBS for 3 days. Pentameric FLAG-Fc protein associated with Myc-IgJ was purified from culture supernatant using an ANTI-FLAG^®^ M2 Affinity Gel (Sigma-Aldrich), and then eluted with glycine-HCl pH 3.0, followed by neutralisation with 1 M Tris-HCl pH 8.5. Protein was concentrated using Amicon Ultra filter concentrators (Millipore, MA), and stored at 4 °C in PBS. Protein concentration was determined by a BCA assay according to the manufacturer’s protocol (Pierce, Rockford, IL).

### Immunoprecipitation and deglycosylation

Five microlitres of cat plasma were incubated with 10 μL anti-feline AIM monoclonal antibody (clone #33) conjugated with protein G Sepharose (GE Healthcare Life Sciences, PA) at 4 °C overnight. The precipitates were washed 5 times with a wash buffer (1% NP-40 in PBS containing protease inhibitors) and eluted in 40 μL glycine-HCl pH 2.0 followed by neutralisation with 1 M Tris-HCl pH 8.5. Deglycosylation was performed using an Enzymatic Protein Deglycosylation Kit (Sigma-Aldrich) following the manufacturer’s protocols. The samples were heated at 95 °C for 5 min with 2 × SDS sample buffer containing 2-mercaptoethanol, and loaded on an SDS-PAGE for immunoblotting.

### Analysis of AIM and IgM levels

Feline AIM in cat plasma and AIM felinised mouse serum were measured by immunoblotting using an anti-feline AIM rabbit polyclonal serum (PAC-11). The lower limit of quantification assessed using feline rAIM as a standard was 2.5 ng/mL for cat AIM. All ELISA assays were performed in a duplicated manner. Cat urinary AIM was measured by ELISA using mouse anti-cat AIM monoclonal antibodies (mouse IgG, clones #33 and #56; generated in our laboratory). The lower limit of quantification assessed by using recombinant AIM protein as a standard was 1.9531 ng/mL for cat AIM. For feline IgM, a Cat IgM ELISA Quantification Set (Bethyl Laboratories, Inc., Montgomery, USA) was used. To analyse feline AIM by ELISA, the urine samples were diluted at 1/10. To analyse plasma IgM, the samples were diluted at 1/3000.

### *In vitro* phagocytosis assay

An *in vitro* phagocytosis assay was performed as described previously^22^. Briefly, to prepare dead cell debris, mProx24 cells, a murine renal proximal tubular epithelial cell line derived from C57BL/6J adult mouse kidney^59^ and kindly provided by Dr Takeshi Sugaya (CMICS Co., Tokyo), were heat-killed by incubation at 65 °C for 20 min in PBS. The dead cells were then labelled with Fixable Viability Dye (FVD, eBioscience) eFluor^®^ 780 followed by being crushed by pipetting vigorously. The fluorescence-labelled dead cell debris were coated with AIM proteins by incubating in serum-free DMEM/F12 (1:1) containing none (control), mouse AIM, or feline AIM (3-SRCR or 4-SRCR) at a concentration of 100 μg/mL at 37 °C for 1 h. To prepare KIM-1-expressing epithelial cells, mProx24 cells were transfected with a mouse or feline KIM-1-IRES-EGFP or EGFP expression vector as a control. At 24 h after transfection, the cells were mixed with the fluorescence-labelled AIM protein-coated dead cell debris as described above in serum-free DMEM/F12 (1:1) supplemented with 5 μg/mL insulin, 5 μg/mL transferrin, and 5 ng/mL selenous acid for 30 min at 37 °C. After incubation, the cells were harvested, washed 3 times with ice-cold PBS, resuspended in PBS containing DAPI, and analysed by flow cytometry (LSRII; BD). Live mProx24 cells were identified as DAPI-negative and the cells overexpressing mouse or feline KIM-1 were determined by EGFP expression. The proportion of engulfment of eFluor^®^ 780 (incorporated dead cell signal)-positive dead cell debris in DAPI-negative EGFP-positive mProx24 cells is presented.

### Generation of AIM felinised mice

A 7-kb mouse genomic DNA fragment of the *AIM (cd5l*) gene promoter region was isolated from C57BL/6 mouse tail DNA by PCR. The promoter fragment and a feline AIM cDNA fragment were inserted into a rabbit β-globin non-coding exon/intron cassette. After removing the vector sequence, the resulting DNA fragment was injected into the prenuclei of C57BL/6 mouse fertilised eggs to produce transgenic mice. The feline AIM transgenic mice were crossbred onto AIM^*−*^ mice to generated AIM felinised mice.

### Statistical analysis

A power calculation was not performed to predetermine sample size. We did not use randomisation to determine samples or mice to be allocated to experiments. Assessment of ATN scores was performed in a blind fashion. *In vitro* experiments were repeated at least three times. Data were analysed using BellCurve for Excel (Social Survey Research Information Co., Ltd.) and are presented as mean values ± s.e.m. unless otherwise specified. Paired results were assessed using parametric tests such as Student’s t-test. Comparisons between multiple groups were analysed using two-way analysis of variance followed by Bonferroni’s post-hoc test. For the Kaplan-Meier curves, *P* values were determined with the log-rank test. Unless otherwise specified, the significance code was added to each figure legend.

## Additional Information

**Accession codes:** The feline AIM (*cd5l*) mRNA sequences have been deposited in the DDBJ/EMBL/GenBank under accession numbers LC149874 (3-SRCR variant 1), LC149875 (3-SRCR variant 2), LC149876 (4-SRCR variant 1) and LC149877 (4-SRCR variant 2).

**How to cite this article**: Sugisawa, R. *et al*. Impact of feline AIM on the susceptibility of cats to renal disease. *Sci. Rep.* **6**, 35251; doi: 10.1038/srep35251 (2016).

## Supplementary Material

Supplementary Information
