# Supplementary material for: Impact of feline AIM on the susceptibility of cats to renal disease
Source: Sci Rep. 2016 Oct 12;6:35251. doi: 10.1038/srep35251 (PMC5059666; doi:10.1038/srep35251)
Supplement: Supplementary Information [file srep35251-s1.pdf]

Supplementary Information for

Impact of feline AIM on the susceptibility of cats to renal disease

Ryoichi Sugisawa, Emiri Hiramoto, Shigeru Matsuoka, Satomi Iwai, Ryosuke Takai, Tomoko Yamazaki, Nobuko Mori, Yuki Okada, Naoki Takeda, Ken-ichi Yamamura, Toshiro Arai, Satoko Arai, Toru Miyazaki

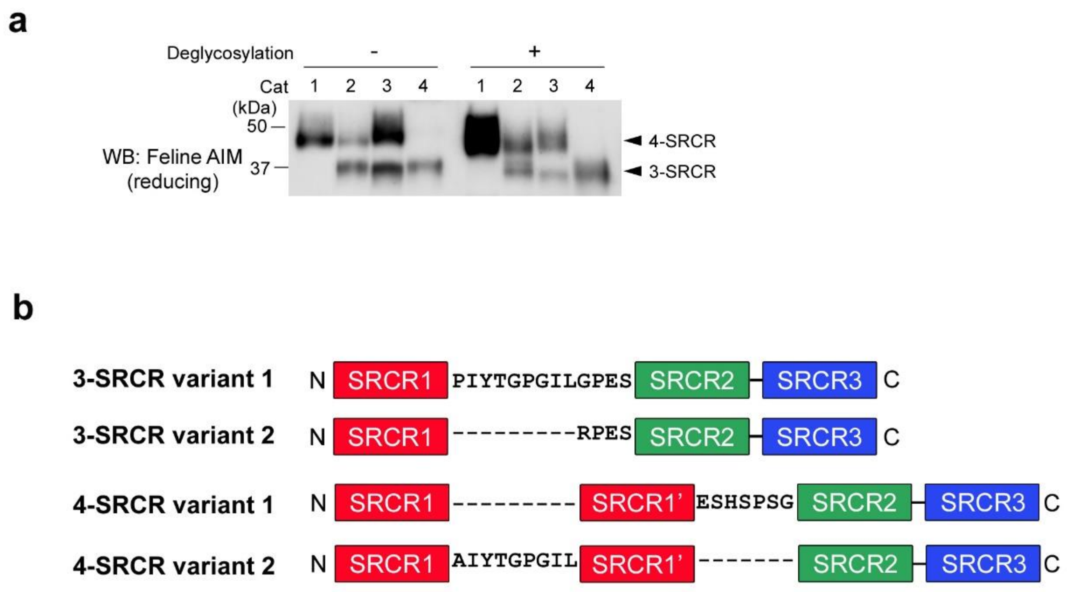

Supplementary Fig. 1. Deglycosylation treatment of feline sera.

(**a**) Serum AIM were immunoprecipitated from cats harboring each AIM-type (#1: 4-/4-SRCR homozygote; #2, 3: 3-/4-SRCR heterozygote; #4: 3-/3-SRCR homozygote) using an anti-feline AIM monoclonal antibody (clone #33). The precipitates with (+) or without (-) deglycosylation using a cocktail of enzymes (PNgase F, O-Glycosidase and α-2(3,6,8,9)-Neuraminidase), were analyzed by immunoblotting in a reducing condition. (**b**) Schemas of feline AIM variants. Two types of 3-SRCR and 4-SRCR feline AIM were identified, in which the variations are present in hinge regions. In both 3-SRCR and 4-SRCR feline AIM, variant 1 is dominant; seventeen out of eighteen 3-SRCR homozygous cats analysed had the variant 1 type 3-SRCR, and three out of four 4-SRCR homozygous cats possessed the variant 1 type 4-SRCR. The precise amino acid sequences are available at GenBank with accession numbers LC149874 (3-SRCR variant 1), LC149875 (3-SRCR variant 2), LC149876 (4-SRCR variant 1) and LC149877 (4-SRCR variant 2).

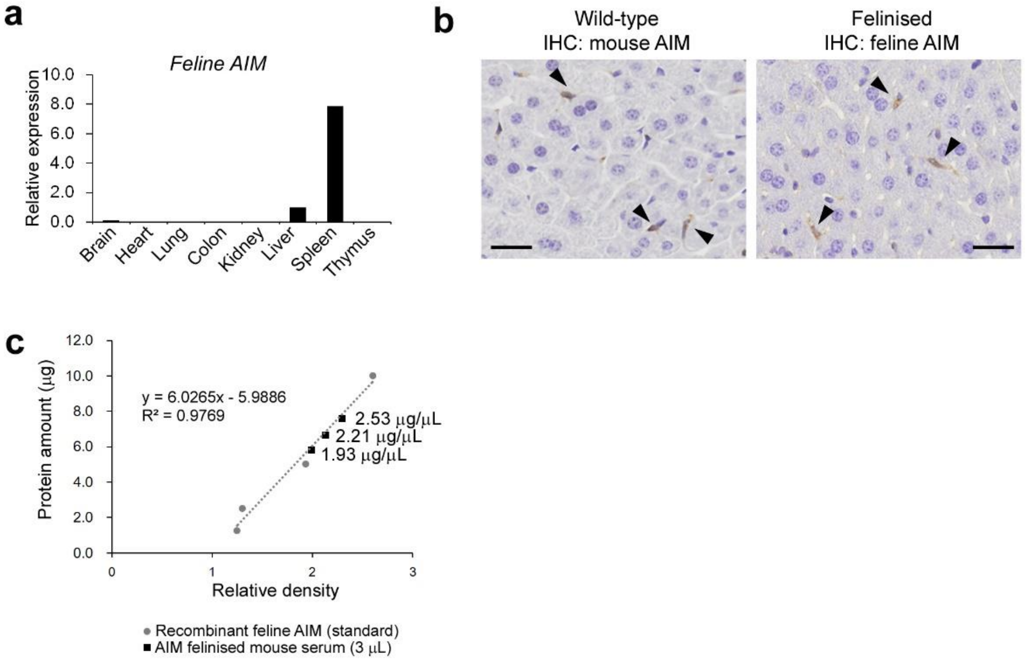

Supplementary Fig. 2. Feline AIM expression in AIM felinised mice.

(**a**) The mRNA levels of the transgenic feline *AIM* in various tissues of AIM felinised mice were analyzed by QPCR. Feline *AIM* mRNA levels relative to that in the liver (of AIM felinised mice) is demonstrated. Three mice were analysed and similar results were obtained. Representative data is presented. (**b**) Feline AIM protein in the liver Kupffer cells of AIM felinised mice analysed by immunohistochemistry (right). Mouse AIM expression in wild-type mouse is presented as a control (left). The arrows indicates AIM signals. Representative photomicrographs out of three mice each, in which similar results were obtained. (**c**) Quantitation of serum feline AIM levels in AIM felinised mice. Serum from three AIM felinsed mice (3 μL from each) were quantified by immunoblotting using recombinant feline AIM protein at different concentrations as quantitative controls. The intensity of AIM signals was evaluated using NIH ImageJ software, and the overall serum AIM concentrations were identified using the standard curve calculated by the signal intensity of control recombinant AIM.

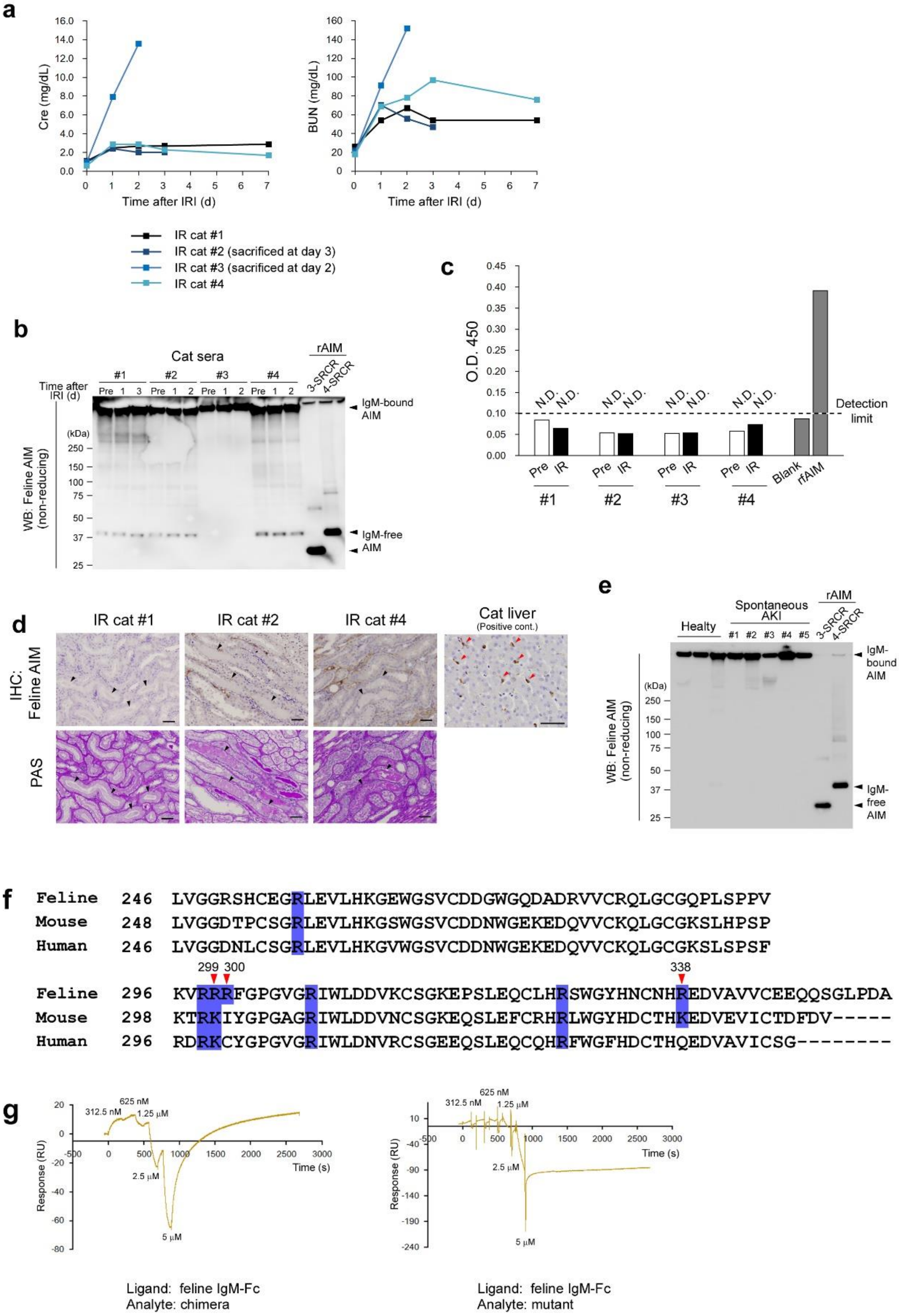

a
Cre (mg/dL)
Time after IRI (d)
BUN (mg/dL)
Time after IRI (d)
IR cat #1
IR cat #2 (sacrificed at day 3)
IR cat #3 (sacrificed at day 2)
IR cat #4
b
Cat sera
rAIM
#1 #2 #3 #4
Time after IRI (d)
Pre 1 3 Pre 1 2 Pre 1 2 Pre 1 2
3-SRCR 4-SRCR
WB: Feline AIM (non-reducing)
(kDa)
250 150 100 75 50 37 25
IgM-bound AIM
IgM-free AIM
c
O.D. 450
N.D.
Detection limit
Pre IR Pre IR Pre IR Pre IR Blank rfAIM
#1 #2 #3 #4
d
IR cat #1
IR cat #2
IR cat #4
Cat liver
(Positive cont.)
IHC: Feline AIM
PAS
e
Healty
Spontaneous AKI
#1 #2 #3 #4 #5
rAIM
3-SRCR 4-SRCR
WB: Feline AIM (non-reducing)
(kDa)
250 150 100 75 50 37 25
IgM-bound AIM
IgM-free AIM
f
Feline 246 LVGGRSHCEGRLEVLHKGEWGSVCDDGWGQDADRVVCRQLGCGQPLSPPV
Mouse 248 LVGGDTPCSGRLEVLHKGSWGSVCDDNWGEKEDQVVCKQLGCGKSLHPSP
Human 246 LVGGDNLCSGRLEVLHKGVWGSVCDDNWGEKEDQVVCKQLGCGKSLSPSF
299 300 338
Feline 296 KVRRRFGPGVGRIWLDDVKCSGKEPSLEQCLHRSWGYHNCNHREDVAVVCEEQQSGLPDA
Mouse 298 KTRKIYGPGAGRIWLDDVNCSGKEQSLEFCRHRLWGYHDCTHKEDVEVICTDFDV-----
Human 296 RDRKCYGPGVGRIWLDNVRCSGEEQSLEQCQHRFWGFHDCTHQEDVAVICSG--------
g
Response (RU)
Time (s)
312.5 nM
625 nM
1.25 μM
2.5 μM
5 μM
Ligand: feline IgM-Fc
Analyte: chimera
Ligand: feline IgM-Fc
Analyte: mutant

Supplementary Fig. 3. Unique characteristics of feline AIM.

(**a**) Levels of Cre and BUN in cats after IR treatments. (**b**) A longer exposure blot for Fig. 3c. Note that a small amount of IgM-free 4-SRCR AIM was detected in #1, 2 (3-/4-SRCR heterozygote genotype) and #4 (4-SRCR homozygote genotype) in pre-IR condition, but its level did not increase after IR. (**c**) Urine samples from cats before (pre) and day1 after IR (IR) were analysed for the presence of AIM using ELISA. The O.D. values of all urine samples were under the detection limit. The O.D. values of the blank control and the recombinant feline AIM (rfAIM, 3.1275 ng/mL) are presented as negative and positive controls, respectively. (**d**) Immunohistostaining (IHC) for feline AIM (upper panels) and PAS staining (lower panels) of the corticomedullary junction area in the kidney of IR-cats #1 (day 7 after IR), 2 (day 3), and 4 (day7). Signals were visualized by HRP/DAB. Scale bars, 50 μm. No obvious AIM staining was observed at the intraluminal debris. Black arrows: intraluminal debris. Immunostaining for feline AIM at the liver specimen of cat is presented as a positive control (upper right panel). Red arrows: feline AIM signals at liver Kupffer cells. (**e**) Immunoblotting for AIM in a non-reducing condition involving sera from 3 healthy cats, and 5 cats with spontaneous AKI. The primary causes for AKI are presented in Extended Data Table 3. (**f**) Alignment of amino acids for SRCR3 of feline, mouse and human AIM. Residues that compose the cluster of the positively charged amino acid in feline AIM, and the corresponding residues in mouse and human AIM, are marked in blue. In mutant feline AIM, feline-specific arginine residues (indicated by red arrows) were substituted to the corresponding amino acids in mouse AIM. (**g**) SPR sensorgrams of chimeric (lef) and mutant (right) feline AIM. Both proteins showed uncontrollable decrease in the binding response, which was caused by a high non-specific binding to the reference flow cell. The strong absorption of both chimeric and mutant feline AIM proteins onto the CM5 sensor chip could not be prevented by blocking procedures either by bovine serum albumin or ethanolamine.

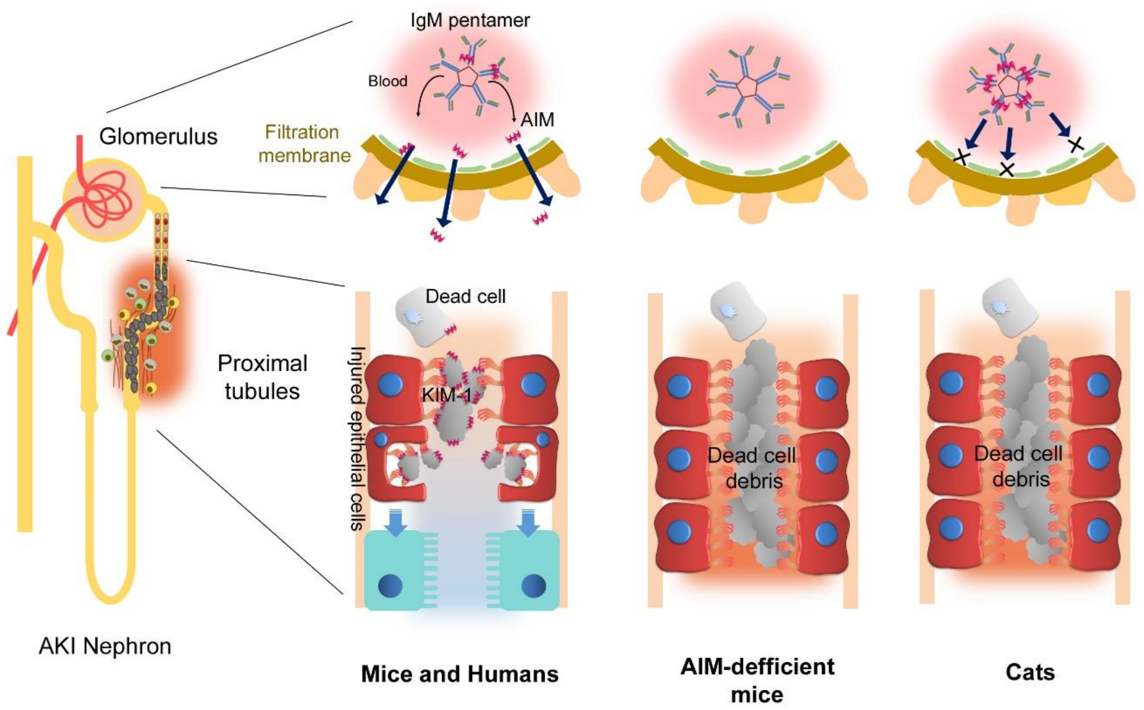

Supplementary Fig. 4 Schema for the involvement of feline AIM in AKI pathogenesis.

In mice and humans, AIM dissociates from IgM pentamer during AKI and is filtrated into urine, enhancing the KIM-1 dependent clearance of lumen-obstructing necrotic cell debris by injured proximal tubular epithelial cells (indicated by red cells), thereby promoting regeneration of epithelial cells (indicated by blue cells) and AKI recovery (left). In AIM-deficient mice, the clearance of intraluminal debris is abrogated (center). In cats, due to the high affinity of feline AIM and IgM, AIM is unable to dissociate from IgM during AKI, abolishing its excretion in urine, and thus, intraluminal debris cannot be cleared (right).

Age	Sex	Breed	AIM type	AIM (μg/mL)
0y 6m	F	Mixed	3-/4-SRCR	22.73
0y 6m	F	Mixed	3-/4-SRCR	15.25
0y 6m	M	Mixed	4-SRCR	22.91
0y 7m	M	Mixed	3-SRCR	14.72
0y 7m	F	Mixed	3-/4-SRCR	10.54
0y 8m	M	Mixed	3-SRCR	16.97
0y 8m	M	Mixed	3-SRCR	10.27
0y 8m	F	Mixed	3-/4-SRCR	14.08
2y 3m	M	Mixed	3-/4-SRCR	23.74
3y 4m	F	Munchkin	3-SRCR	23.29
3y 4m	M	Somali	4-SRCR	23.62
4y 0m	M	Mixed	3-/4-SRCR	19.98
4y 2m	M	Mixed	3-SRCR	12.85
4y 5m	M	Mixed	3-SRCR	24.87
5y 6m	F	Mixed	4-SRCR	20.32
5y 8m	M	Turkish angora	4-SRCR	27.01
6y 2m	F	Mixed	3-SRCR	23.24
6y 6m	F	Mixed	3-/4-SRCR	17.26
6y 7m	M	Munchkin	3-SRCR	8.48
7y 1m	F	Mixed	3-SRCR	30.88
7y 4m	F	Mixed	3-SRCR	26.93
7y 5m	F	Mixed	3-SRCR	36.39
7y 6m	F	Mixed	3-/4-SRCR	27.80
7y 6m	M	Mixed	4-SRCR	23.98
7y 9m	M	Chinchilla	4-SRCR	27.81
9y 8m	M	Mixed	3-SRCR	22.49
11y 6m	M	Mixed	3-/4-SRCR	24.33
11y 8m	F	Mixed	3-SRCR	31.21
12y 6m	F	American shorthair	3-/4-SRCR	15.89
13y 0m	F	Mixed	3-/4-SRCR	30.16
14y 2m	M	American curl	3-/4-SRCR	15.37
14y 4m	F	Mixed	4-SRCR	29.60

Supplementary Table 1. Serum AIM levels in cats.

Serum AIM levels of 32 (16 females and 16 males; average age: 5 years 11 months) cats were analysed by immunoblotting. The average serum AIM level was 21.72 ± 6.78 μg/mL. Age, sex, breed (strain), and AIM type of them are also presented.

No.	Age	Sex	Breed	AIM type
1	5 years	Female	Mixed	3-/4-SRCR
2	8 years	Female	Mixed	3-/4-SRCR
3	9 years	Male	Mixed	3-SRCR
4	7 years	Male	Mixed	4-SRCR

Supplementary Table 2. Age, Sex, breed, and AIM type of the 4 cats subjected to IR.

No.	Cre (mg/dL)	Age	Sex	Breed	AIM type	Cause(s) of AKI
1	4.18	3 years	Male	Scottish fold	3-SRCR	Ureteric stone
2	6.21	2 years	Male	Norwegian forest cat	3-/4-SRCR	Ureteric stone, urethra stone
3	6.82	5 years	Male	Somali	3-SRCR	Ureteric stone
4	7.52	12 years	Male	American shorthair	3-/4-SRCR	Ureteric stone
5	7.80	Unknown	Female	Chartreux	3-SRCR	Kidney stone

Supplementary Table 3. Clinical information (Cre levels and causes of AKI), as well as age, sex, breed and AIM type of five with spontaneous AKI.

	Name	Sequence (5' - 3')
QPCR	f-feline GAPDH	AAGGGTGGGGCCAAGAGG
	r-feline GAPDH	AGAGGGGCCAGGCAGTTG
	f-feline KIM-1	ACCTCAAGCCAGCTACTTCATCTT
	r-feline KIM-1	TGGTGTCCTCGTCTCCTGTG
	f-feline AIM	TCAAGTGCTCGGGGAAGGAG
	r-feline AIM	TCTTCACAGACCACAGCCACA
	f-mouse GAPDH	AGAACATCATCCCTGCATCC
	r-mouse GAPDH	CACATTGGGGGTAGGAACAC
Cloning	fSRCR1_rev	AACTCCACACGACCTTCACAGCGG
	fAIM_5_f_EcoRI	GCGGAATTCTCGCCTGGCTCATGGCGCTACTCT
	fAIM_3_r_XhoI	GCGCTCGAGAGCATCAGGTAGGCCAGACTGCTG

Supplementary Table 4. Sequences of oligonucleotides used in QPCR and cloning of feline AIM cDNA.
